# An immune-related lncRNA risk coefficient model to predict the outcomes in clear cell renal cell carcinoma

**DOI:** 10.18632/aging.203797

**Published:** 2021-12-26

**Authors:** Cheng Tang, GenYi Qu, Yong Xu, Guang Yang, Jiawei Wang, Maolin Xiang

**Affiliations:** 1 Department of Urology, The Affiliated Zhuzhou Hospital XiangYa Medical College CSU, Zhuzhou 412007, China

**Keywords:** TCGA database, clear cell renal cell carcinoma, immune-related lncRNA pairs, risk coefficient model, prognosis, tumor immune infiltration, targeted therapy

## Abstract

Objective: Using model algorithms, we constructed an immune-related long non-coding RNAs (lncRNAs) risk coefficient model to predict outcomes for patients with clear cell renal cell carcinoma (ccRCC) to understand the infiltration of tumor immune cells and the sensitivity to immune-targeted drugs.

Methods: Open genes data were downloaded from The Cancer Genome Atlas and The Immunology Database and Analysis Portal, and immune-related lncRNAs were obtained through Pearson correlation analysis. R language software was used to obtain differentially expressed immune-related lncRNAs and immune-related lncRNA pairs. The model was constructed using least absolute shrinkage and selector operation regression analysis, and receiver operator characteristic curves were drawn. The Akaike information criterion was used to distinguish the high-risk from the low-risk group. We also conducted correlation analysis for the high- and low-risk subgroups.

Results: We identified 27 immune-related lncRNAs pairs, 16 of which were included in the model construction. After merging clinical data, the areas under the curve of 1 -year, 3-year, and 5-year survival times of ccRCC patients were 0.867, 0.832, and 0.838, respectively. Subgroup analyses were conducted according to the cut-off value. We found that the high-risk group was associated with poor outcomes. The risk score and tumor stage were independent predictors of the outcome of ccRCC. The risk model predicted specific immune cell infiltration, immune checkpoint gene expression levels, and high-risk groups more sensitive to sunitinib targeted therapy.

Conclusion: We obtained prognostic-related novel ccRCC markers and risk model that predicts the outcome of patients with ccRCC and helps identify those who can benefit from sunitinib.

## INTRODUCTION

Renal cancer is among the top ten most common cancers worldwide; it accounts for 5% of new cases each year. Approximately 45,520 people were diagnosed with renal cancer in the United States in 2020 [1]. Clear cell renal cell carcinoma (ccRCC) is the most common subtype of renal cancer, accounting for about 85% of the total cases, with a male-to-female ratio of 1.7:1. Most patients are middle-aged and elderly, with an average age of 64 [2].

Surgery is the standard treatment for localized ccRCC; however, this cancer carries a high risk of metastasis, poor outcome and is insensitive to conventional chemotherapies and radiotherapies [3]. In the most recent decade, with the rapid development of molecular biological tools, drug treatment of ccRCC ranged from the initial non-specific immunotherapy to targeted therapy and then to the immune checkpoint inhibitors [4]. Among them, PD-1-based blocking therapy has become the first-line therapy for patients with advanced or platinum-intolerant ccRCC [5–7]. In the phase III trial CheckMate 025, nivolumab was more effective than everolimus in treating patients with advanced ccRCC who had previously received treatment (the 5-year survival rate was about 26% vs. 18%). The (Food and Drug Administration) FDA approved Nivolumab for the treatment of ccRCC [8]. Although some of these drugs can improve outcomes, their effectiveness is limited [9]. Therefore, we need to explore the mechanisms of ccRCC occurrence and development and identify new biomarkers to predict related drug sensitivity.

The tumor microenvironment of ccRCC is heterogeneous, and several studies showed that the degree of immune cell infiltration in the tumor microenvironment was related to outcomes. Studies showed that high levels of CD8+ T cell infiltration in ccRCC was associated with poor outcome. Macrophages were also an essential part of the tumor microenvironment; high degrees of infiltration of M2-type macrophages are associated with tumor invasion and poor outcome of ccRCC [5]. Therefore, it is necessary to identify immune-related tumor prognostic markers.

Long non-coding RNA (lncRNA) does not code for protein. The length is more than 200 bp. It was initially considered a by-product of RNA polymerase II transcription and did not have biological functions. Many studies showed that lncRNA has a conserved secondary structure that can interact with proteins, DNA and RNA, and participate in regulating various biological processes, especially in tumors. It plays critical regulatory roles in tumors, including chromatin modification, transcription activation and inhibition, post-transcriptional mediation, and miRNA-induced molecular interference with gene expression [6–8]. Many studies showed that changes in molecular biology are closely related to the occurrence and development of ccRCC. Transcriptome studies described the abnormal expression of specific long non-coding RNAs, and the occurrence and progression of ccRCC have a close relationship. The expression level of lncRNA HOTAIRM1 in ccRCC decreased and inhibited the hypoxic pathway of tumor development [10]. Another study showed that lncRNA URRCC and EGFL7/P-AKT/FOXO3 signaling was related to poor outcomes and promoted the proliferation and invasion of ccRCC [11]. Another study showed that lncARSR transported by exosomes promoted the expression of AXL and c-MET in ccRCC cells by competitively binding miR-34/miR-449, rendering ccRCC patients resistant to sunitinib [12]. The binding lncRNA-LET and miR-373-3p induced the up-regulation of DKK1 and TIMP2 levels and reduced the anti-tumor effect of lncRNA-LET-mediated by ccRCC cells [13]. Other studies demonstrated that immune-related lncRNA has vital clinical significance in predicting outcomes in patients with ccRCC and as a target for targeted therapy [14–19]. Therefore, the present study aimed to construct an immune-related lncRNA risk coefficient model using a model algorithm, lncRNA pairing, and iteration to predict outcomes in patients with ccRCC, understanding the tumor immune cell infiltration and the sensitivity of targeted drugs.

## RESULTS

**Figure 1:**
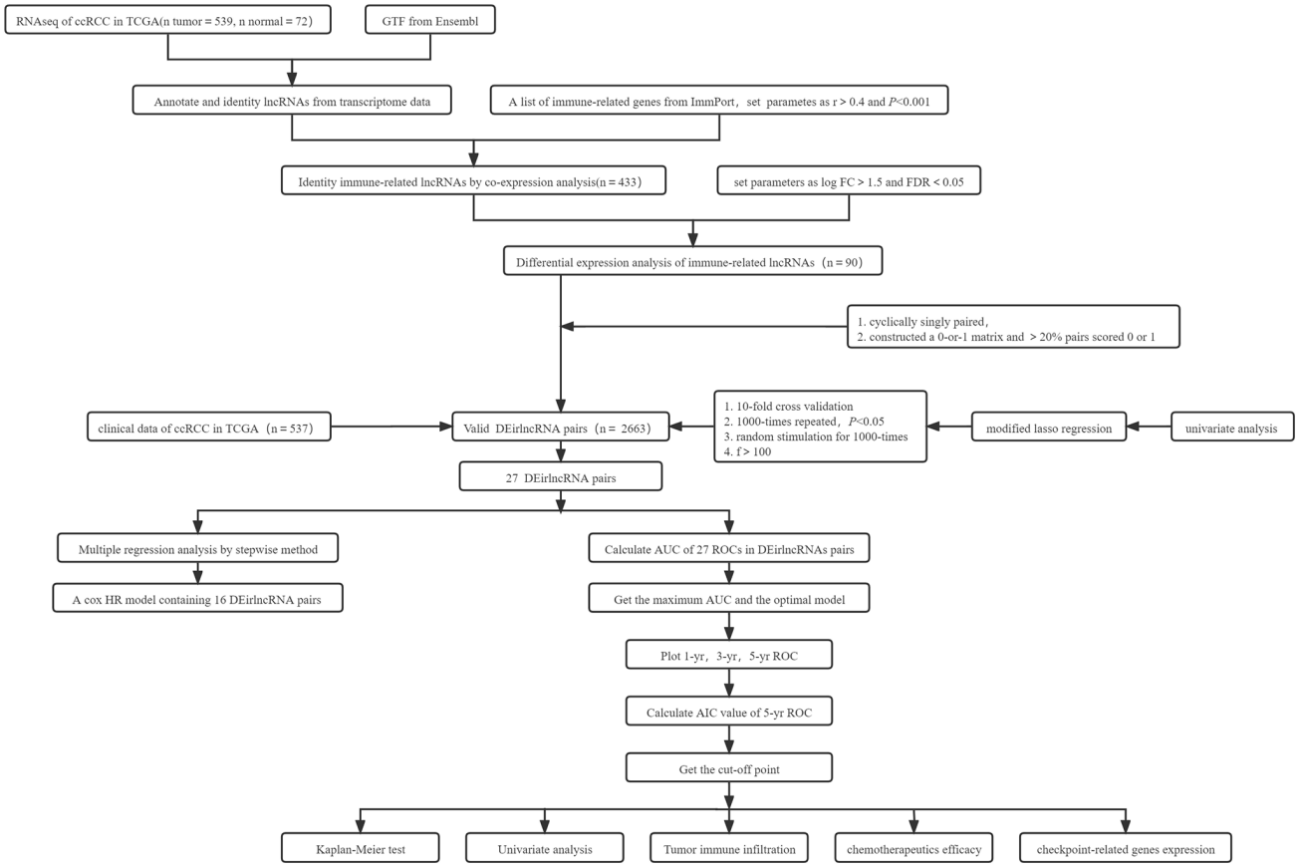
Summary flowchart of this study.

The illustration of summary highlight was provided in Figure 1.

### Analysis of differential expression of immune-related lncRNAs in ccRCC

**Figure 2:**
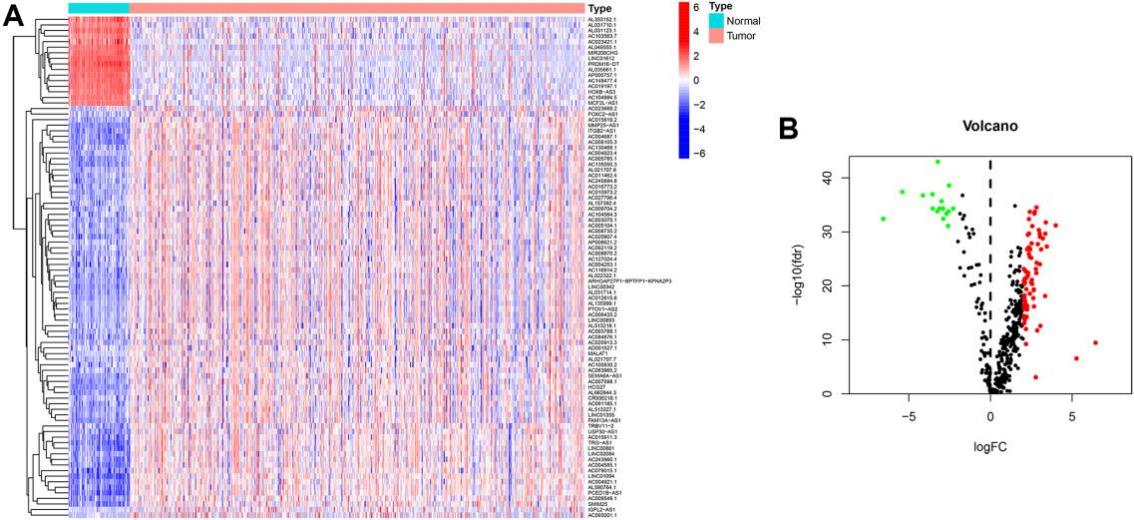
**Heatmap and differential expression analysis of immune-related lncRNA in ccRCC.** (**A**) Heatmap of immune-related lncRNA genes between clear cell renal cell carcinoma and normal tissues. Red indicates upregulated, and blue indicates downregulated. (**B**) Volcano map of immune-related lncRNA between clear cell renal cell carcinoma and normal tissues. Red dots: upregulation with significant differential expression, green dots: downregulation with significant differential expression, black dots indicate no significant difference.

The transcriptome and immune gene-related data of ccRCC were obtained from The Cancer Genome Atlas (TCGA) database and The Immunology Database and Analysis Portal (ImmPort). The Ensembl database was used to annotate and distinguish transcriptome data. Using Pearson correlation analysis, with co-expression correlation coefficient >0.4 and *P* < 0.001 as the identifying criteria, 433 immune-related lncRNAs were identified. We used differential expression analysis, with |log Fold Change| >1.5 and false discovery rate (FDR) <0.05 as the identifying criteria. We obtained 90 differentially expressed immune lncRNAs, and the gene heatmap (Figure 2A) was generated using R software. Sixteen lncRNAs expressions were downregulated, and 74 lncRNAs were upregulated in ccRCC (Figure 2B and Table 1).

**Table 1:** Immune-related lncRNAs of ccRCC obtained after differential expression analysis.

**lncRNA**	**Normal-mean**	**Tumor-mean**	**logFC**	***P*-value**	**FDR**
AC104984.5	3.682574	0.462925451	−2.99186	3.54E-38	2.04E-36
AC015911.3	0.100497	0.821778506	3.0316	7.92E-31	8.18E-30
PTOV1-AS2	0.642868	2.771483237	2.108063	1.53E-18	5.01E-18
AC003070.1	0.283999	1.390484124	2.291631	2.55E-23	1.27E-22
AC020913.3	0.074073	0.617973411	3.060524	1.27E-13	2.62E-13
AD001527.1	0.127594	0.576003883	2.174521	4.91E-13	9.69E-13
AC004687.1	0.090107	0.986754328	3.452974	5.85E-29	5.24E-28
AC007098.1	0.106151	0.572507398	2.431175	2.41E-28	1.87E-27
AC093001.1	0.011144	0.965969528	6.437666	2.11E-10	3.40E-10
AC103563.7	5.173696	0.597977578	−3.11303	1.51E-36	4.93E-35
HOXB-AS3	5.586956	0.94096728	−2.56985	6.02E-36	1.62E-34
AL021707.6	0.457954	1.920309814	2.068065	1.75E-18	5.68E-18
AL590764.1	0.15393	0.753471696	2.291277	3.26E-31	3.46E-30
SMIM25	0.25703	1.518804932	2.562927	6.13E-33	8.52E-32
TRG-AS1	0.110214	0.712890989	2.69338	1.82E-35	4.08E-34
PRDM16-DT	13.2686	0.316471521	−5.3898	2.97E-40	3.99E-38
TRBV11-2	0.139708	0.8630982	2.627106	5.48E-19	1.87E-18
AL049555.1	3.540425	0.370257526	−3.25732	5.29E-36	1.52E-34
SEMA6A-AS1	0.144724	0.740373342	2.354945	1.72E-27	1.24E-26
AC091185.1	0.171909	0.846735721	2.300265	7.20E-24	3.72E-23
AL021707.7	0.12328	0.576657008	2.225772	5.35E-17	1.43E-16
AL513327.1	0.253827	1.052985268	2.05257	1.35E-24	7.65E-24
LINC00861	0.089858	0.728956024	3.02011	1.93E-31	2.18E-30
AC079015.1	0.069813	0.732073012	3.390413	1.14E-33	1.77E-32
AC084876.1	0.082949	0.572314341	2.78651	1.30E-24	7.46E-24
AC127024.4	0.250425	1.055792994	2.075876	3.21E-17	8.87E-17
AC010973.2	0.170749	0.775083366	2.182473	4.60E-24	2.44E-23
AC023421.1	10.33512	0.109043989	−6.5665	2.25E-34	3.94E-33
ARHGAP27P1-BPTFP1-KPNA2P3	0.317314	1.367695028	2.107763	9.89E-23	4.69E-22
AL513218.1	0.142618	0.595938402	2.063012	4.51E-15	1.02E-14
CR936218.1	0.259281	1.093931575	2.076936	9.57E-22	4.10E-21
PCED1B-AS1	0.477539	3.126144012	2.710694	7.88E-36	1.92E-34
FOXC2-AS1	0.099379	0.724179466	2.865338	9.22E-13	1.79E-12
LINC00893	0.168803	0.800961054	2.246392	9.65E-17	2.51E-16
LINC02084	0.147412	0.832738092	2.498013	9.70E-27	6.51E-26
AC011462.4	0.384346	1.66693917	2.116724	2.50E-18	7.88E-18
AL031710.1	10.23424	1.697657813	−2.59179	5.68E-33	8.17E-32
AC004921.1	0.13765	0.704239881	2.355067	2.66E-34	4.28E-33
AC008735.2	0.609316	3.237210057	2.409489	7.61E-20	2.71E-19
MCF2L-AS1	2.411878	0.498237863	−2.27525	1.59E-36	4.93E-35
AL662844.3	0.115057	0.989206462	3.103922	2.20E-28	1.74E-27
IGFL2-AS1	0.021451	0.827439957	5.269534	2.01E-07	2.79E-07
AC023669.2	0.277618	1.903775796	2.777691	0.000737	0.000869
AL135999.1	0.211246	0.893068672	2.079847	7.64E-17	2.00E-16
AP000757.1	8.871118	1.183177983	−2.90645	1.56E-36	4.93E-35
AL022322.1	0.196393	1.37638469	2.809066	8.38E-24	4.28E-23
AC104564.3	0.177313	0.780916828	2.138872	7.16E-18	2.15E-17
AC004253.1	0.148081	0.831291733	2.488964	5.64E-22	2.47E-21
USP30-AS1	0.394299	2.072622237	2.394094	1.15E-28	9.68E-28
LINC01355	0.246017	1.126133673	2.19455	1.08E-20	4.17E-20
AC004923.4	0.157851	0.649031036	2.039724	3.03E-22	1.41E-21
AC019197.1	2.144548	0.331330829	−2.69433	2.06E-35	4.31E-34
FAM13A-AS1	0.216505	1.241791946	2.519949	1.42E-29	1.33E-28
AC093788.1	0.138316	0.584969989	2.080399	3.62E-19	1.25E-18
HCG27	0.202245	1.578450713	2.964331	3.72E-32	4.68E-31
AC016773.2	0.107834	0.677946827	2.652365	1.23E-20	4.70E-20
AC092119.2	0.153225	0.65949918	2.105717	8.33E-15	1.86E-14
AC148477.4	5.974009	0.803677607	−2.89401	2.37E-34	3.98E-33
AC005785.1	0.121053	0.610457023	2.334251	9.01E-29	7.89E-28
AC027796.4	0.224249	1.066627441	2.24988	1.74E-17	4.93E-17
AC245884.8	0.222197	1.305749985	2.554966	1.03E-21	4.38E-21
AC005104.1	0.290904	1.230954211	2.081162	1.63E-19	5.75E-19
AC009549.1	0.273816	1.404515616	2.358792	1.63E-31	1.99E-30
AP006621.2	0.441335	1.854161375	2.07082	3.74E-14	8.01E-14
AC100830.2	0.236398	0.996619494	2.075825	1.22E-17	3.56E-17
LINC01612	3.150561	0.338202929	−3.21965	2.61E-46	1.05E-43
AL353152.1	2.991248	0.17047375	−4.13313	2.50E-39	1.75E-37
AL035661.1	29.66428	2.555782522	−3.53689	1.54E-36	4.93E-35
AC012615.6	0.180508	0.82261601	2.188153	7.62E-23	3.65E-22
MALAT1	15.83501	71.62380448	2.177322	3.77E-10	5.96E-10
AL031123.1	10.58834	0.906174056	−3.54654	1.09E-39	1.09E-37
AC135050.3	0.160602	1.482246649	3.206223	1.68E-30	1.65E-29
AC006435.2	0.201304	0.937788214	2.219884	2.41E-15	5.55E-15
LINC01094	0.27725	1.963631257	2.824262	6.62E-37	2.96E-35
AC008870.2	0.140459	0.60869582	2.11557	5.25E-22	2.33E-21
AC009704.2	0.150205	0.940764575	2.646897	1.69E-20	6.36E-20
AC243960.1	0.190792	0.90584361	2.24726	2.19E-28	1.74E-27
AC008105.3	0.051766	0.825717921	3.995577	4.20E-33	6.28E-32
AC130469.1	0.057492	0.584918994	3.346804	2.14E-19	7.51E-19
AC116914.2	0.23694	1.374503075	2.536315	1.69E-22	7.93E-22
AL031714.1	0.188508	0.798453427	2.082584	1.83E-24	1.03E-23
AC020907.4	0.134378	1.094545179	3.025967	1.54E-25	1.00E-24
MMP25-AS1	0.315379	1.680744965	2.41394	8.09E-36	1.92E-34
AC063965.2	0.111037	0.705899432	2.668423	2.30E-17	6.49E-17
AL157392.4	0.134044	0.566137711	2.078443	1.63E-15	3.77E-15
ITGB2-AS1	0.223036	1.665270736	2.900407	2.01E-29	1.84E-28
AC015819.2	0.090653	0.645324543	2.831606	8.37E-26	5.53E-25
LINC00342	0.591509	3.152218235	2.413896	6.71E-27	4.58E-26
AC004585.1	0.089846	0.922131089	3.359442	2.00E-31	2.18E-30
MIR200CHG	2.760587	0.476473591	−2.53451	1.27E-41	2.56E-39

Abbreviations: logFC: log fold change; FDR: false discovery rate.

### Establishment of differentially expressed immune lncRNA pairs and risk coefficient scoring model

**Figure 3:**
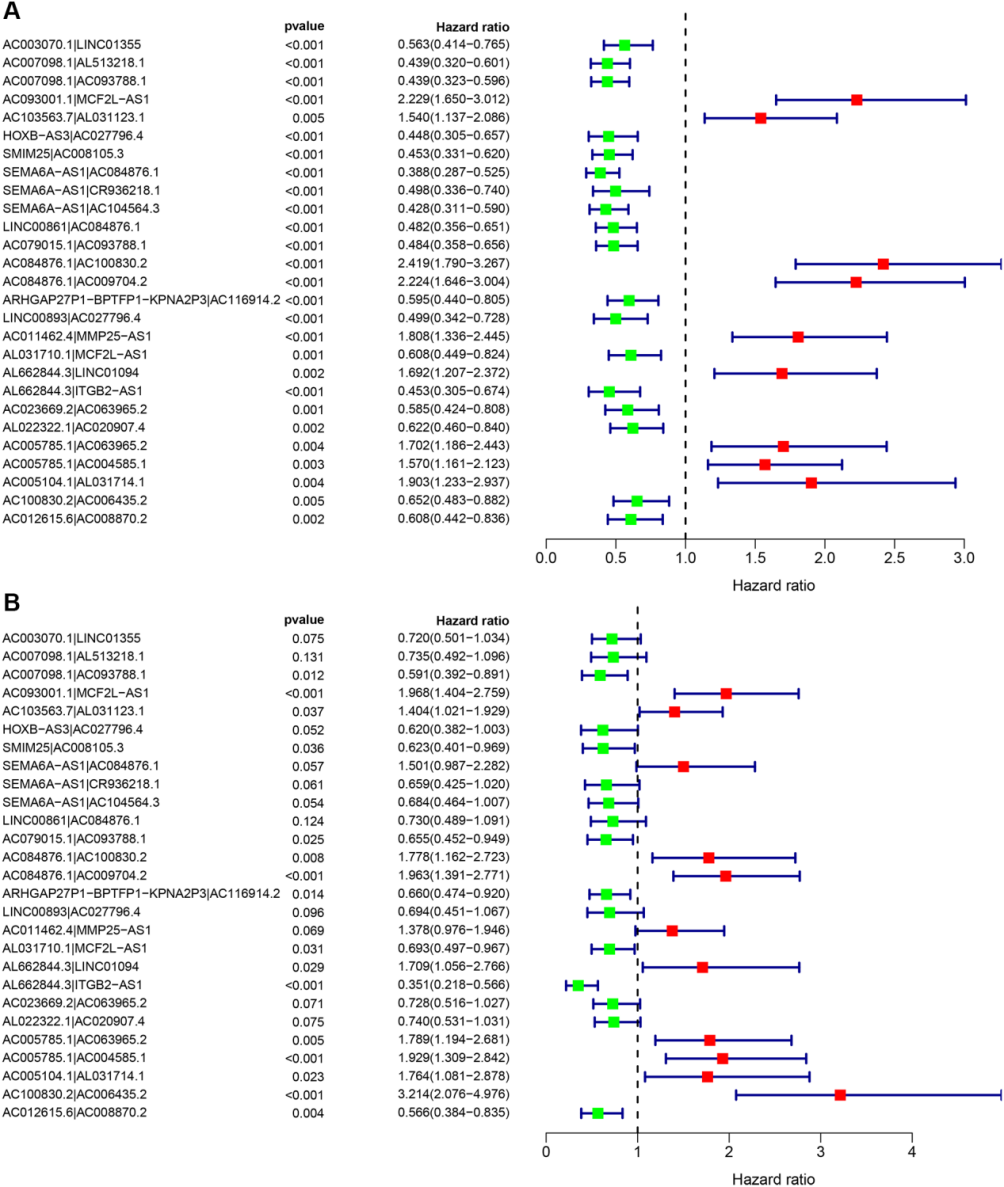
**Cox regression analysis was performed on 27 immune lncRNA pairs related to clear cell renal cell carcinoma outcome.** (**A**) Cox univariate regression analysis forest plot of 27 immune lncRNA pairs related to the outcome of clear cell renal cell carcinoma. (**B**) Cox multivariate regression analysis forest plot of 27 immune lncRNA pairs related to clear cell renal cell carcinoma outcome. Red indicates risk factors, and green indicates protective factors.

By matching the 90 differentially expressed immune lncRNA pairs for multiple cycles, a total of 2663 differentially expressed immune lncRNA pairs were obtained. Next, 27 immune lncRNA pairs were identified using least absolute shrinkage and selector operation (LASSO) regression analysis and Cox univariate regression analysis (Figure 3A). Then, a Cox multivariate regression analysis was performed based on these 27 immune lncRNA pairs, 16 of which can participate in constructing the risk coefficient scoring model (Figure 3B) and the risk coefficient of each immune lncRNA pair was obtained (Table 2).

**Table 2:** Analysis of regression coefficients of 27 pairs of immune-related lncRNA to Cox related to outcome.

**lncRNA pairs**	**Coefficient**	**HR**	**HR.95L**	**HR.95H**	***P*-value**
AC003070.1|LINC01355	−0.3288	0.719785	0.500928	1.034261	0.075434
AC007098.1|AL513218.1	−0.30849	0.734555	0.492259	1.09611	0.13089
AC007098.1|AC093788.1	−0.52659	0.590616	0.391629	0.890708	0.012002
AC093001.1|MCF2L-AS1	0.677176	1.968312	1.404197	2.759052	8.49E-05
AC103563.7|AL031123.1	0.339006	1.403552	1.021037	1.929371	0.03678
HOXB-AS3|AC027796.4	−0.47882	0.619511	0.382477	1.003444	0.051656
SMIM25|AC008105.3	−0.47275	0.623284	0.40096	0.968881	0.035692
SEMA6A-AS1|AC084876.1	0.406183	1.501078	0.987318	2.282178	0.0574
SEMA6A-AS1|CR936218.1	−0.41778	0.658509	0.425095	1.020089	0.06136
SEMA6A-AS1|AC104564.3	−0.38017	0.683744	0.464403	1.006684	0.054078
LINC00861|AC084876.1	−0.31441	0.730217	0.488926	1.090588	0.124475
AC079015.1|AC093788.1	−0.42386	0.654517	0.451517	0.948784	0.025254
AC084876.1|AC100830.2	0.575764	1.778489	1.161522	2.723171	0.008078
AC084876.1|AC009704.2	0.67463	1.963306	1.390942	2.771194	0.000125
ARHGAP27P1-BPTFP1-KPNA2P3|AC116914.2	−0.41481	0.660465	0.474051	0.920182	0.014223
LINC00893|AC027796.4	−0.3658	0.693642	0.450763	1.06739	0.096231
AC011462.4|MMP25-AS1	0.320517	1.37784	0.975717	1.945689	0.068706
AL031710.1|MCF2L-AS1	−0.36667	0.693035	0.496767	0.966848	0.030895
AL662844.3|LINC01094	0.53578	1.70878	1.055544	2.766279	0.029265
AL662844.3|ITGB2-AS1	−1.04623	0.351258	0.21793	0.566157	1.74E-05
AC023669.2|AC063965.2	−0.31732	0.728096	0.516044	1.027285	0.070809
AL022322.1|AC020907.4	−0.30121	0.739923	0.531166	1.030724	0.074908
AC005785.1|AC063965.2	0.581787	1.789233	1.194042	2.681107	0.004812
AC005785.1|AC004585.1	0.656907	1.928817	1.309024	2.842067	0.000895
AC005104.1|AL031714.1	0.567601	1.76403	1.081279	2.877891	0.023033
AC100830.2|AC006435.2	1.167458	3.213814	2.075641	4.976102	1.66E-07
AC012615.6|AC008870.2	−0.56878	0.566217	0.383901	0.835116	0.004121

Abbreviations: HR: hazard ratio; HR.95L: 95% CI lower limit; HR.95H: 95% CI upper limit.

### Evaluation of the prognostic predictive power of the risk model

**Figure 4:**
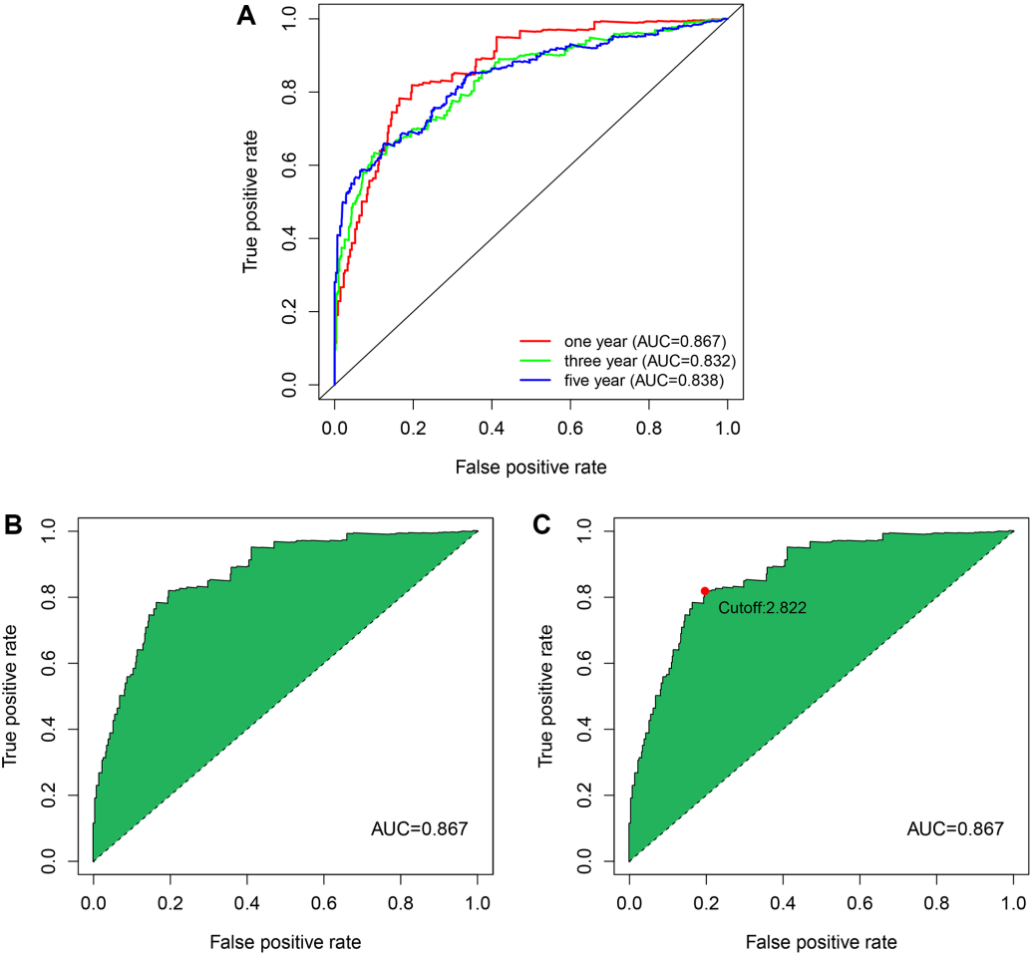
**The ROC curves by establishing the risk coefficient model through the immune lncRNA pairs of ccRCC.** (**A**) The 1, 3, and 5-year ROC curves were obtained using model construction. The AUC values were all higher than 0.83. (**B**) One-year ROC curve with maximum AUC value obtained by the model. (**C**) The cut-off value of 2.822 that distinguishes between high- and low-risk patients was obtained using the best fit.

Above 27 prognostic-related immune lncRNA pairs were used to construct the 1-year, 3-year, and 5-year receiver operator characteristic (ROC) curves of patients (Figure 4A), and the 1-year area under the curve (AUC) was calculated to be the largest AUC of 0.867 (Figure 4B). In addition, the 3-year and 5-year AUC obtained were 0.832 and 0.838, respectively, which also had predictive power. Through the best fit, the cut-off value for distinguishing between high-and low-risk groups of ccRCC patients was 2.822. We included 190 patients in the low-risk group and 360 patients in the high-risk group.

### Analysis of the correlation of clinical indicators by the risk model

**Figure 5:**
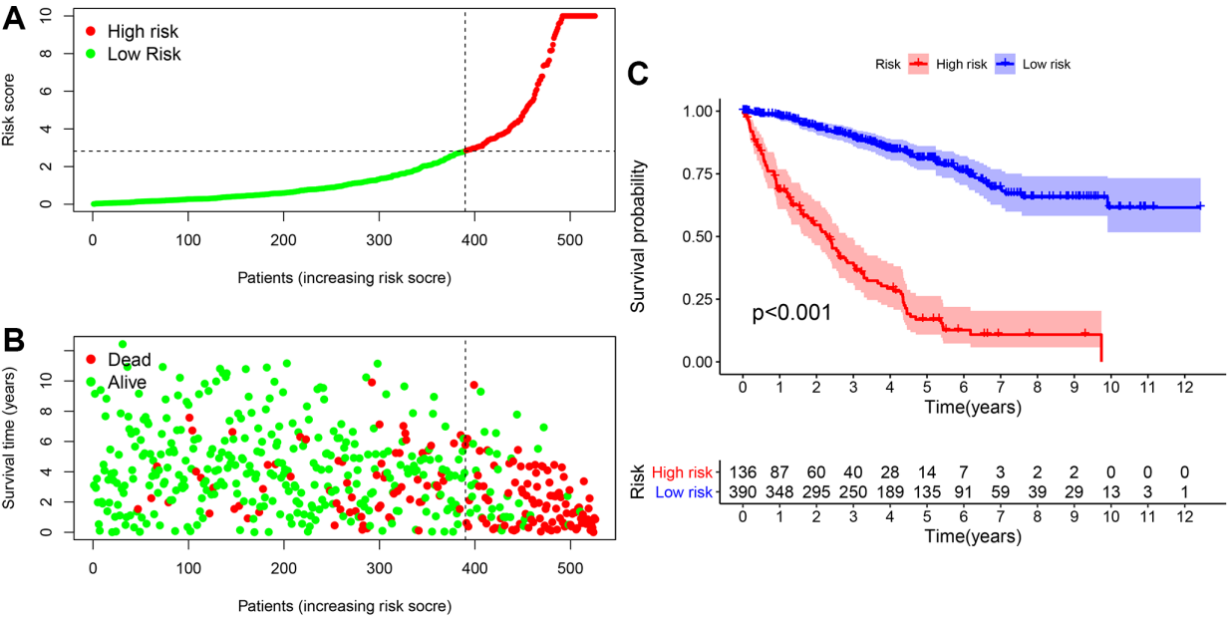
**The risk coefficient model of ccRCC predicted outcome.** (**A**) The risk score was divided into high- and low-risk groups. (**B**) Scatter plot of risk score and outcome for each patient. (**C**) A Kaplan-Meier curve was constructed based on the survival status of the high- and low-risk groups.

The relationship between the risk factor score and the risk subgroup patients ccRCC was analyzed via R language software (Figure 5A). According to the time process, the relationship between the patient’s survival status and the risk coefficient score were obtained (Figure 5B), and the Kaplan-Meier curve was constructed according to the survival status of the high- and low-risk groups (Figure 5C). The result was that the survival rate of patients in the low-risk group was significantly higher than that in the high-risk group (*P* < 0.001).

**Figure 6:**
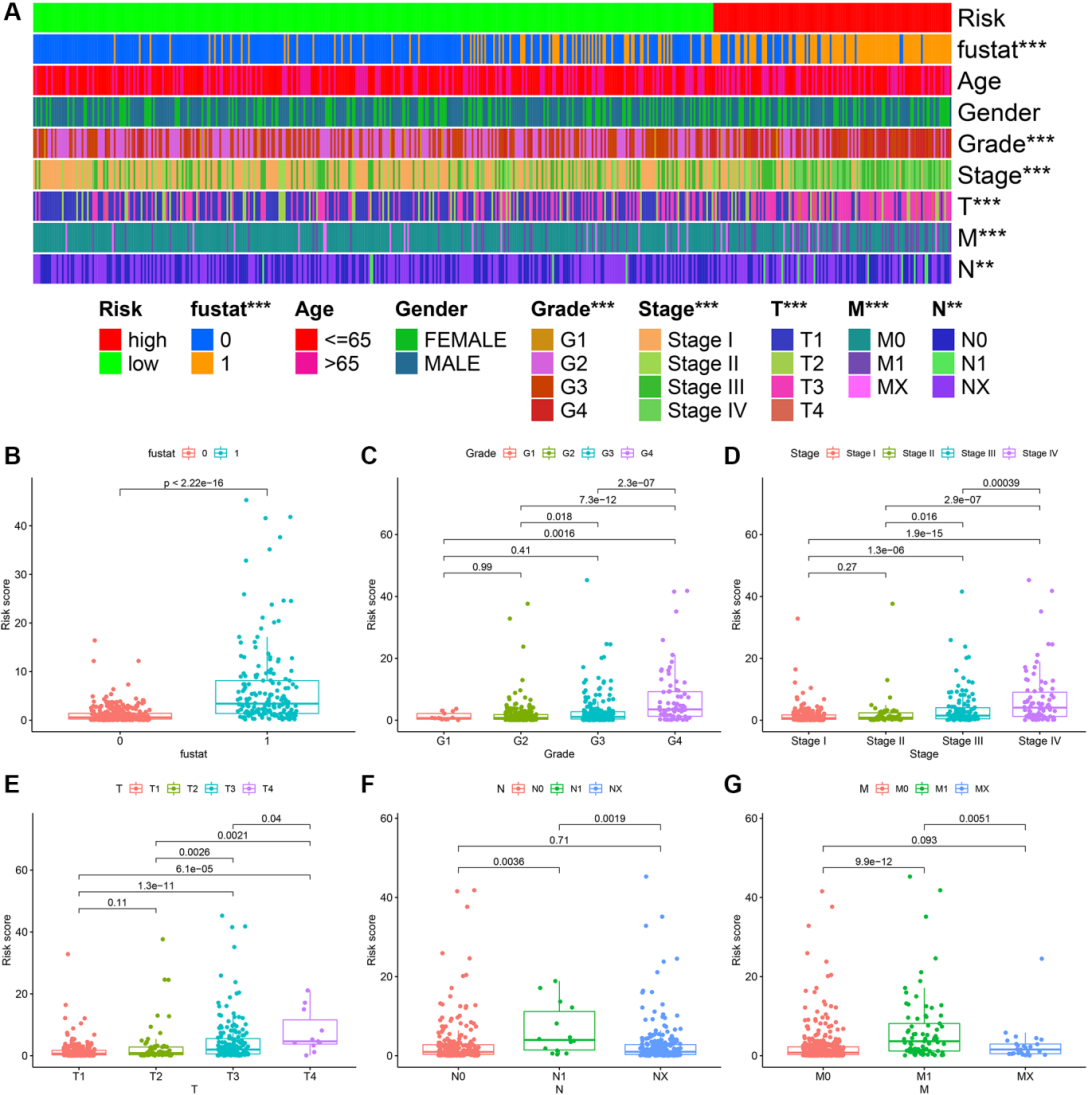
**ccRCC risk coefficient model for clinical correlation analysis.** The clinical correlation heatmap (**A**) illustrating that survival (**B**), tumor grade (**C**), tumor clinical stage (**D**), T stage (**E**), N stage (**F**), and M stage (**G**) were closely related to risk factor scores.

The heatmap in Figure 6A described the relationship between the level of risk scores and clinically relevant indicators. We found that the survival status of patients with ccRCC (*P* < 0.001), tumor grade (*P* < 0.001), tumor stage (*P* < 0.001), T stage (*P* < 0.001), M stage (*P* < 0.001), and N stage (*P* < 0.01) were related to risk coefficient score significantly. It can be seen from the box plot we constructed that ccRCC patients with a higher risk factor had a higher chance of death (Figure 6B). Furthermore, tumor grade (Figure 6C), tumor clinical stage (Figure 6D), T stage (Figure 6E), N stage (Figure 6F), and M stage (Figure 6G) were also higher.

**Figure 7:**
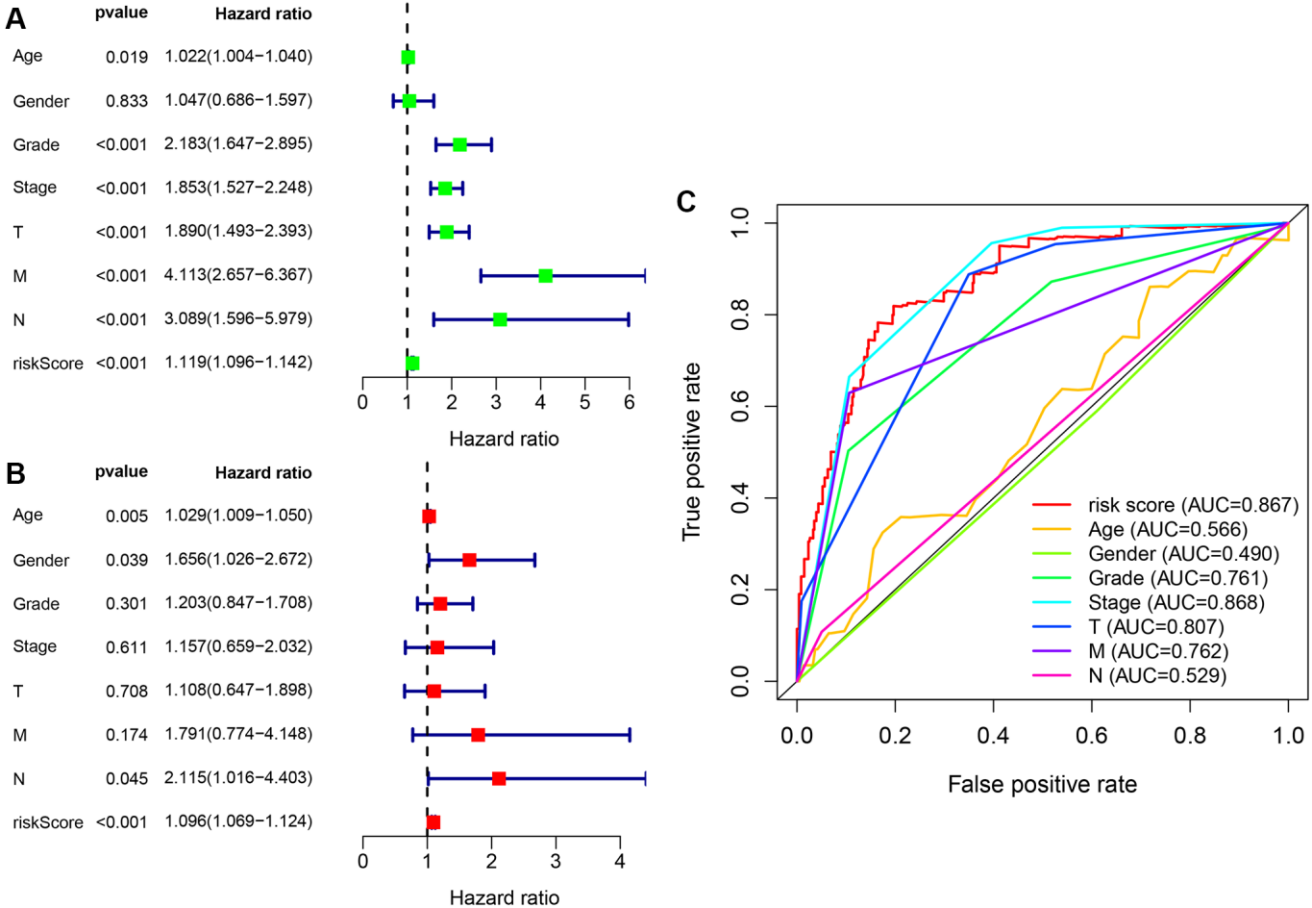
**Cox regression analysis of clinical correlation indicators and integrated ROC curves.** (**A**) Clinical-related indicators Cox univariate regression analysis showing that tumor grade, clinical stage, TMN stage, and risk score were related to outcome. (**B**) Cox multivariate analysis showing that risk scores are independent predictors of outcome. (**C**) The comparison of risk coefficient score and clinical-related indicators showing that risk coefficient score (AUC = 0.867) and tumor stage (AUC = 0.868) had the highest predictive efficacy.

A Cox univariate and a multivariate regression analysis were performed on the risk score and clinical correlation indicators. Then the R language software’s survival package was used to visualize the data, and forest maps were done (Figure 7A and Figure 7B). It was found that the tumor grade, tumor stage, TNM stage, and risk coefficient score were related to the outcome of the Cox univariate analysis, but in the Cox multivariate analysis, age, gender, and risk coefficient score were independent predictors of outcome. The ROC curve of clinical-related indicators and the 1-year risk coefficient score were compared in the same chart (Figure 7C). The result was that the patient’s risk coefficient score (AUC = 0.867) and tumor stage (AUC = 0.868) had the highest predictive efficacy.

### Correlation analysis between risk coefficient model and immune cell infiltration

**Figure 8:**
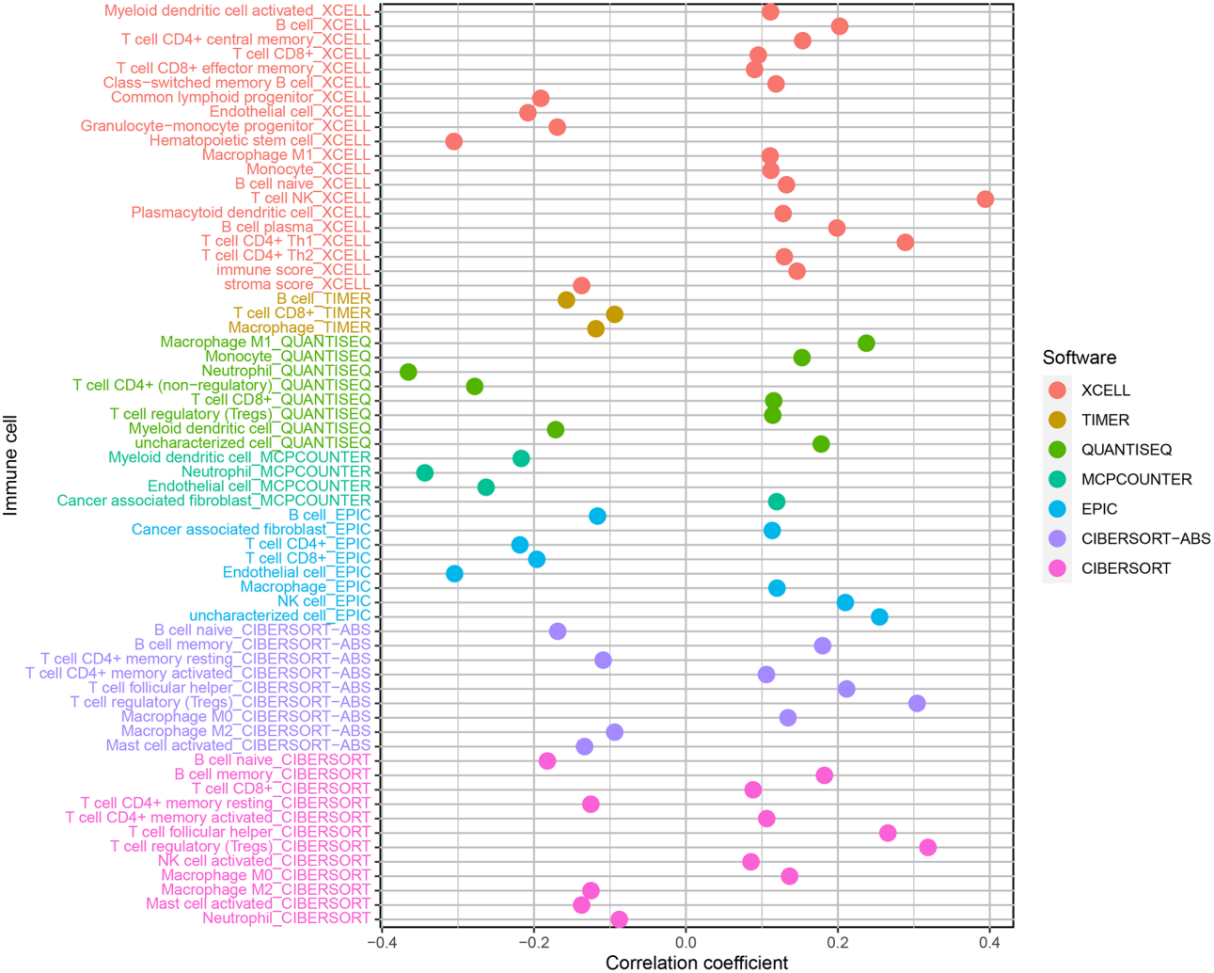
Correlation analysis of immune infiltrating cells in ccRCC.

XCELL, TIMER, QUANTISEQ, MCPCOUNTER, EPIC, CIBERSORT-ABS, and CIBERSORT were used to estimate the proportion of immune cells in these samples of ccRCC patients based on marker gene and deconvolution algorithm. Pearson correlation test was used to analyze the correlation between the risk coefficient model and tumor immune infiltrating cells with screening criteria *P* < 0.05, and R language software was used for data visualization (Figure 8). We found that the samples of the high-risk group were positively correlated with the infiltration of NK cells, regulatory T cells, and M1 macrophages in ccRCC and negatively correlated with the infiltration of neutrophils in ccRCC.

### Correlation analysis of risk coefficient model with genes

**Figure 9:**
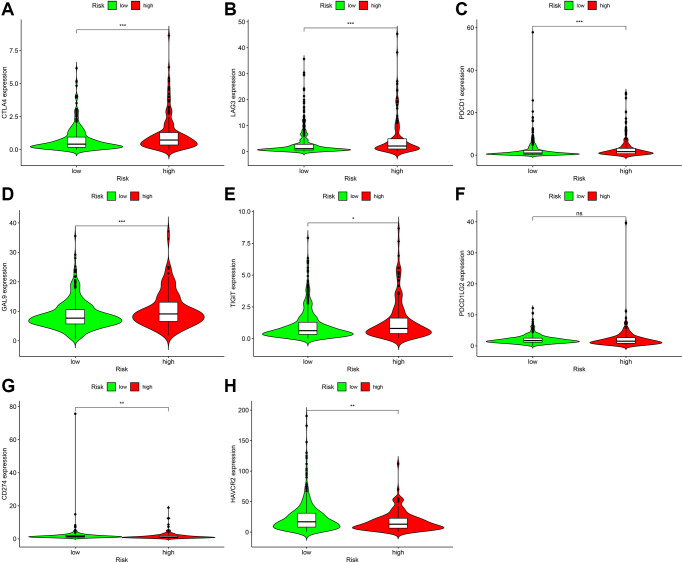
**Correlation analysis of genes in patients with ccRCC.** In the high-risk group, the expression levels of CTLA4 (**A**), LAG3 (**B**), PDCD1 (**C**), GAL9 (**D**), and TIGIT (**E**) increased. Although the expression level of PDCD1LG2 (**F**) increased, it was not statistically significant. CD274 (**G**) The expression level of HAVCR2 (**H**) decreased.

Immune-targeted therapy is one of the most popular drugs for the treatment of renal clear cell carcinoma. We further explored the relationship between the risk coefficient model and genes, and found that among the high-risk patients, the expression levels of CTLA4 (*P* < 0.001; Figure 9A), LAG3 (*P* < 0.001; Figure 9B), PDCD1 (*P* < 0.001; Figure 9C), GAL9 (*P* < 0.001; Figure 9D), and TIGIT (*P* < 0.001; Figure 9E) increased. The expression level of PDCD1LG2 increased but not significantly (*P* > 0.05; Figure 9F). The expression levels of CD274 *(P* < 0.01; Figure 9G), HAVCR2 (*P* < 0.01; Figure 9H) decreased in this model. These genes are the potential therapeutic targets for ccRCC.

### Correlation analysis between risk coefficient model and targeted therapy drugs

**Figure 10:**
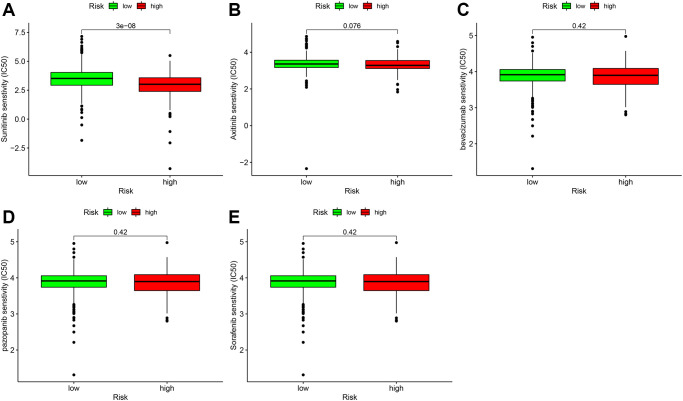
**Correlation analysis of immune-targeted drugs in patients with ccRCC.** The risk factor score was used as a potential predictor. Compared with the low-risk group, the sunitinib IC50 value of high-risk patients was lower (**A**), which was statistically significant (*P* = 3e-08). Axitinib (**B**), bevacizumab (**C**), and pazopanib (**D**), and sorafenib (**E**) were not significantly different between the high- and low-risk groups.

Targeted therapy drugs are the first-line therapy for patients with advanced ccRCC. We also analyzed the relationship between the risk coefficient scoring model and the sensitivity of targeted therapy drugs. The IC_50_ was used to evaluate the efficacy of drugs. Lower IC_50_ suggests higher sensitivity. We found that the high-risk group was associated with higher sensitivity of sunitinib (Figure 10A), which was statistically significant (*P* = 3 e-08); axitinib (Figure 10B), bevacizumab (Figure 10C), pazopanib (Figure 10D), and sorafenib (Figure 10E) were not significantly different in the high- and low-risk groups.

## DISCUSSION

Many studies found that transcriptome RNA expression levels are related to the outcomes of malignant tumors [20–22]. Recent research on the role of non-coding RNA in ccRCC is also a focus of research [10, 11, 13, 23]. In previous studies, lncRNA-related models of ccRCC were constructed based on the expression level of transcriptome data [14, 16, 24]. In the present study, we used immune-related lncRNAs pairs to construct risk coefficient models to assess the outcome of patients with ccRCC, not based on expression levels of lncRNA. We first used TCGA and ImmPort to obtain the lncRNA and immune-related gene data of patients with ccRCC and then used R software to identify immune-related lncRNAs. Then, the differential expression of ccRCC and normal samples adjacent to the cancer were analyzed, and immune-related lncRNA pairs were obtained. We obtained the risk coefficient of each sample of ccRCC patients and established a risk coefficient model using Cox univariate factors, multivariate regression analysis, and LASSO regression analysis. By generating ROC curves, we found that the AUC for one-year outcome was the largest, and the Akaike information criterion (AIC) optimal fitting was used to obtain the critical value for distinguishing high from low-risk groups. The survival analysis of the high- and low-risk groups showed that the survival rate of patients in the low-risk group was significantly higher than that of the high-risk group (*P* < 0.001).

We also calculated the correlation between the risk coefficient score of each ccRCC sample and various clinical indicators. We found that age, gender, and risk coefficient score were independent predictors of outcome through Cox multivariate regression analysis. We also constructed the ROC curve of clinical-related indicators, which compared the ROC curve of the 1-year risk coefficient score in the same chart. We found that the one-year outcome risk coefficient score and tumor stage were the best predictors of ccRCC outcome, suggesting the reliability of the risk coefficient model.

To analyze the relationship between risk factor score and immune cell infiltration, the immune cell infiltration data of patients with ccRCC, we used XCELL, TIMER, QUANTISEQ, MCPCOUNTER, EPIC, CIBERSORT-ABS, and CIBERSORT and correlation analysis. We found that the level of infiltration of NK cells, regulatory T cells, and M1 macrophages in the high-risk group was high.

Sierra et al. found that tumor-infiltrating PD-L1+ NK cells were highly expressed in renal clear cell carcinoma patients. In *in vitro* experiments, NK cells inhibited the proliferation of CD8+ T cells, suggesting that NK tumors infiltrating cells weakened immune regulatory functions [25]. A study showed that high levels of infiltration of dendritic cell quiescence, dendritic cell activation, mast cell quiescence, mast cell activation, and eosinophils were associated with a good outcome in ccRCC patients, while B cell memory, T cell follicular helper cells, and T cell regulation associated with poor outcome in ccRCC [26]. Xu et al. found that HK3 promoted the infiltration of monocytes and macrophages that present cell surface antigens and regulated the critical genes PD-1 and CTLA-4 of debilitating T cells, thereby affecting the immune escape process [27].

We also performed correlation analysis on the risk model for immune checkpoint genes and targeted therapy drugs and found that expression levels of CTLA4, LAG3, PDCD1, GAL9, and TIGIT increased, while expression levels of CD274 and HAVCR2 decreased in samples from patients in the high-risk group; these can be used as immune targeted therapy with potential therapeutic targets.

ccRCC discovered early and mid-term can be removed surgically, while patients with advanced ccRCC experience poor outcomes because of metastasis and missing the optimal time for surgery. Immune-targeted drug therapy has become the first-line treatment for patients with advanced ccRCC. Of these, vascular endothelial growth factor monoclonal antibody and tyrosine kinase inhibitors are the primary drugs used for anti-tumor angiogenesis therapy [28–30]. However, changes in the tumor microenvironment may be associated with the emergence of resistance of ccRCC to immune-targeted drugs. Therefore, identifying sensitive drugs may reduce treatment costs and reduce the side effects of immune-related drugs. In the risk coefficient model, we included sunitinib, axitinib, bevacizumab, pazopanib, and sorafenib and found that patients with ccRCC in the high-risk group were more sensitive to sunitinib than the low-risk group.

This study has some limitations, although we adopted rigorous methods and algorithms to build the model. It is necessary to validate the reliability of our risk coefficient model using external data. In a future study, we will collect more clinical data and expand the sample size.

Outcome-related novel ccRCC markers and risk coefficient model were obtained by constructing immune-related lncRNA pairs, which can predict outcomes of ccRCC and help distinguish those who can benefit from sunitinib.

## MATERIALS AND METHODS

### Data acquirement

The GDC Data Transfer Tool was used to download open transcriptome data from TCGA (https://cancergenome.nih.gov/) [31] of ccRCC and normal tissues adjacent to the cancer, which included 539 cases of ccRCC samples and 72 cases of normal tissue samples adjacent to cancer. Then gene transfer format (GTF) files were downloaded using Ensembl (http://asia.ensembl.org) [32] to annotate and distinguish the mRNA and lncRNA of the transcriptome data. Immune-related genes were obtained from ImmPort (http://www.immport.org).

### Differential expression analysis of immune-related lncRNAs

Immune-related lncRNAs were identified based on the co-expression strategy and Pearson correlation analysis according to the co-expression correlation coefficient > 0.4 and *P* < 0.001. DEirlncRNA was selected using the limma package of R language software [33] with |log Fold Change|>1.5 and FDR < 0.05. Obtained lncRNAs were visualized using the heatmap package.

### Construction of immune-related lncRNA pairs

DEirlncRNAs were identified using multiple rounds of pairing. Parameter values of 0 or 1 were used for definition, and α was defined as the parameter value. If the expression of lncRNA A was greater than that of lncRNA B in an immune-related lncRNA pair in a sample, then the α value of the lncRNA pair was 1; otherwise, the value of α was 0. If the ratio of the α value (either 0 or 1) of the immune-related lncRNA pair in all samples is less than 80%, it means that the immune-related lncRNA pair was an effective match; otherwise, it needs to be re-paired.

### Acquisition of clinical data and establishment of model

First, clinical data related to ccRCC were downloaded from TCGA. Then, the limma package of R software was used to match the immune-related lncRNA pairs in the previous step. We then took the intersection and deleted the repeated clinical data with no follow-up time. A single factor regression analysis was performed on the immune-related lncRNA pairs initially obtained, and the immune-related lncRNA pairs related to the survival status were found. The significance screening criterion was *P* < 0.01.

To prevent over-fitting, the glmnet package of R language was used to perform LASSO regression analysis [34] on the obtained immune lncRNA pairs, run 1000-repeated random cycles, and immune lncRNA pairs with a matching frequency of more than 100 times identified those with *P* < 0.05 after the second cross-validation. The best pairing combination was selected to obtain immune lncRNA pairs that can participate in constructing the Cox risk coefficient model. By constructing Cox univariate and multivariate analysis models, the risk coefficient of each immune lncRNA pair related to the outcome was obtained, and the risk score of each patient’s tumor sample was determined. The total risk score of each ccRCC patient sample was equal to the sum of the expression amount of each immune lncRNA pair in the sample multiply risk coefficient. The formula is following:


$$\text{Risk Score}=\sum_{i=1}^{n}{\text{Risk Coefficient}}_{i}\times \text{I}{\text{rlncRNA Expression}}_{i}$$

The Cox analysis results were visualized using the survminer and survival packages of R software.

### Construction of the ROC curve with risk coefficient model

ROC curves were constructed using the survivalROC package of the R software, which included ROC for 1-, 3-, and 5- years and the AUC values were calculated to determine the value predicted by the model. We found that the 1-year ROC curve had the largest AUC value. According to the AIC best fit [35], it was possible to distinguish low-risk with high-risk patients by finding a critical value with the largest sum of specificity and sensitivity.

### Clinical correlation analysis with risk coefficient model

Survival and survminer packages of the R language software were used to compare the survival differences between the high- and low-risk groups. *P* < 0.001 indicated a significant difference. A Kaplan-Meier curve was constructed to visualize the data. The relationships between the risk score and the previously obtained clinical indicators (survival status, age, gender, tumor grade, tumor stage, and T, N, and M stages) were analyzed using the chi-square test. The relationships between the risk score and the different subgroups of clinical indicators were analyzed using the Wilcoxon rank-sum test. The limma package and ggpubr package of the R language software were used to visualize the data. To determine whether the risk score can be used as an independent predictor related to the outcome of patients with ccRCC, we performed Cox univariate and multivariate regression analysis on the risk score and clinical correlation indicators, which used the hazard ratio to evaluate. *P* < 0.05 was the identification criterion, and the survival package of R software was used to visualize the data. To compare the accuracy of the risk score and clinically relevant indicators in predicting survival and outcome, we compared the ROC curves obtained for the 1-year follow-up with the ROC curve of clinically relevant indicators in the same graph.

### Correlation analysis of immune cells

To analyze the relationship between risk factor score and immune cell infiltration, the immune cell infiltration data of patients with ccRCC in TCGA was calculated based on CIBERSORT (http://cibersort.stanford.edu/) [36], TIMER (version 2.0; http://timer.cistrome.org/) [37], QUANTISEQ (http://icbi.at/quantiseq) [38], Microenvironment Cell Populations-counter [39], EPIC (http://epic.gfellerlab.org) [40], and XCELL (http://xCell.ucsf.edu/) [41]. The correlation between immune cell infiltration data and risk coefficient score was analyzed using the limma, scales, ggplot2, and ggtext packages of R software, which can be visualized to obtain a bubble chart according to *P* < 0.05 as the identifying criterion.

### Gene correlation analysis

We found that CD274, CTLA4, HAVCR2, LAG3, LGALS9, PDCD1, PDCD1LG2, and TIGIT were abundantly expressed in ccRCC samples. To determine whether these genes differed between the high- and low-risk groups of the risk model, the limma and ggpubr packages of the R language software were used to analyze and visualize the data using violin charts.

### Correlation analysis of targeted drugs

To determine whether there was a difference in patients’ response in the high- and low-risk groups of ccRCC patients to targeted drugs, the half-inhibition rate (IC_50_) of the drug was used as an index to measure drug sensitivity. The data were analyzed and visualized using the limma package, ggpubr, ggplot, and pRRophetic package in R software.
